# Amyloid-independent atrophy patterns predict time to progression to dementia in mild cognitive impairment

**DOI:** 10.1186/s13195-017-0299-x

**Published:** 2017-09-12

**Authors:** Mara ten Kate, Frederik Barkhof, Pieter Jelle Visser, Charlotte E. Teunissen, Philip Scheltens, Wiesje M. van der Flier, Betty M. Tijms

**Affiliations:** 1 Alzheimer Center & Department of Neurology, Neuroscience Campus Amsterdam, VU University Medical Center, Amsterdam, The Netherlands; 2 Department of Radiology and Nuclear Medicine, Neuroscience Campus Amsterdam, VU University Medical Center, Amsterdam, The Netherlands; 3 Institutes of Neurology and Healthcare Engineering, University College London, London, UK; 4 Department of Psychiatry & Neuropsychology, School for Mental Health and Neuroscience, Maastricht University, Maastricht, The Netherlands; 5 Neurochemistry Laboratory and Biobank, Department of Clinical Chemistry, VU University Medical Center, Neuroscience Amsterdam, Amsterdam, The Netherlands; 6 Department of Epidemiology and Biostatistics, VU University Medical Center, Amsterdam, The Netherlands; 7 Alzheimer Center & Department of Neurology, VU University Medical Center, PO Box 7057, 1007 MB Amsterdam, The Netherlands

**Keywords:** Mild cognitive impairment, Alzheimer’s disease, Magnetic resonance imaging, survival analysis

## Abstract

**Background:**

Amyloid pathology in subjects with mild cognitive impairment (MCI) is an important risk factor for progression to dementia due to Alzheimer’s disease. Predicting the onset of dementia is challenging even in the presence of amyloid, as time to progression varies considerably among patients and depends on the onset of neurodegeneration. Survival analysis can account for variability in time to event, but has not often been applied to MRI measurements beyond singular predefined brain regions such as the hippocampus. Here we used a voxel-wise survival analysis to identify in an unbiased fashion brain regions where decreased gray matter volume is associated with time to dementia, and assessed the effects of amyloid on these associations.

**Methods:**

We included 276 subjects with MCI (mean age 67 ± 8, 41% female, mean Mini-Mental State Examination 26.6 ± 2.4), baseline 3D T1-weighted structural MRI, baseline cerebrospinal fluid (CSF) biomarkers, and prospective clinical follow-up. We fitted for each voxel a proportional Cox hazards regression model to study whether decreased gray matter volume predicted progression to dementia in the total sample, and stratified for baseline amyloid status.

**Results:**

Dementia at follow-up occurred in 122 (44%) subjects over an average follow-up period of 2.5 ± 1.5 years. Baseline amyloid positivity was associated with progression to dementia (hazard ratio 2.4, *p* < 0.001). Within amyloid-positive subjects, decreased gray matter volume in the hippocampal, temporal, parietal, and frontal regions was associated with more rapid progression to dementia (median (interquartile range) hazard ratio across significant voxels 1.35 (1.32–1.40)). Repeating the analysis in amyloid-negative subjects revealed similar patterns (median (interquartile range) hazard ratio 1.76 (1.66–1.91)).

**Conclusions:**

In subjects with MCI, both abnormal amyloid CSF and decreased gray matter volume were associated with future progression to dementia. The spatial pattern of decreased gray matter volume associated with progression to dementia was consistent for amyloid-positive and amyloid-negative subjects.

**Electronic supplementary material:**

The online version of this article (doi:10.1186/s13195-017-0299-x) contains supplementary material, which is available to authorized users.

## Background

Subjects with mild cognitive impairment (MCI) are at increased risk of dementia with annual conversion rates of 10–15% [1, 2]. According to the NIA–AA research criteria [3], a diagnosis of MCI due to AD requires the presence of amyloid pathology as measured in cerebrospinal fluid (CSF) or on amyloid PET. Since amyloid reaches a plateau relatively early in the disease course [4–6], it has limited prognostic value for the time to onset of dementia. Neuronal injury markers, such as brain atrophy measured with structural MRI, are more closely related to cognitive impairment and could thus be useful for estimating time to clinical progression [7–10]. Previous studies found that hippocampal atrophy can be used to predict time to dementia in MCI patients with positive amyloid markers [11, 12]. However, other brain regions may also be valuable for the prediction of progression, as indicated by voxel-based morphometry studies [13–15]. Two previous studies have used hypothesis-free voxel-level survival analyses to show that decreased gray matter in the medial temporal lobe and posterior cingulate cortex can predict time to conversion to AD-type dementia in nondemented subjects [16, 17]. However, it is still unclear whether such predictive atrophy patterns are specific for amyloid pathology.

In this study, we performed unbiased voxel-wise survival analyses to detect brain regions where decreased gray matter volume is associated with time to progression to dementia, and examined whether patterns associated with progression to dementia were dependent on amyloid status in a large sample of subjects with MCI.

## Methods

### Participants

Subjects with a clinical diagnosis of MCI, a good-quality structural MRI and CSF biomarker assessment at baseline, and at least one clinical follow-up were selected from the CODA (COnnectivity in DementiA) study, which includes subjects from the Amsterdam Dementia Cohort [18]. This cohort consists of subjects attending the memory clinic of the VU University Medical Centre Amsterdam since 2000. All subjects in this cohort underwent routine dementia screening, including physical and neurological examination, neuropsychological testing, brain MRI scanning, and usually lumbar puncture (unless contraindication or patient refusal). The study protocol was approved by the VU University Medical Centre institutional review board. All subjects gave written informed consent for their clinical data to be used for research purposes.

### Clinical assessment

Baseline clinical diagnosis of MCI was established during a consensus meeting from a multidisciplinary team according to the Petersen criteria [19]. Subjects were followed annually, and duration of follow-up ranged from 1 to 11 years (mean 2.5 ± 1.5). A follow-up diagnosis was made during a multidisciplinary consensus meeting according to common clinical and research criteria [19–24]. Time to dementia was defined as the time between the baseline visit and the date of dementia diagnosis. The primary analysis included all subjects, regardless of follow-up diagnosis. Analyses were repeated for the subsample of subjects who converted to AD-type dementia.

### CSF analysis

CSF was collected at baseline by lumbar puncture using a 25-gauge needle in polypropylene tubes (Sarstedt, Nümbrecht, Germany). CSF was centrifuged at 1800 × *g* for 10 min at 4 °C and stored at – 20 °C until biomarker analysis, within 2 months after collection. CSF Aβ_1–42_ was measured using InnoTest sandwich ELISAs (Innogenetics, Fujirebio, Ghent, Belgium) [25]. Subjects were classified as amyloid positive or negative with a cut-off point of CSF Aβ_1–42_ < 640 ng/L [26].

### MRI acquisition

Anatomical 3D T1-weighted images were acquired at baseline as part of regular patient care with eight different scanners using a spoiled gradient echo type of sequence (e.g., MPRAGE, FSPGR, TFE). Details of scanners and acquisition parameters can be found in Additional file 1. The MRI protocol also included a 3D fluid attenuated inversion recovery (FLAIR) sequence, dual-echo T2-weighted sequence, susceptibility weighted imaging (SWI), and diffusion weighted imaging (DWI) to visually assess brain pathology by an experienced neuroradiologist.

### MRI analysis

Structural 3D T1 images were segmented using Statistical Parametric Mapping 12 (SPM12) software (Wellcome Trust Centre for Neuroimaging, University College London, UK) running in MATLAB 2011a (MathWorks Inc., Natick, MA, USA). Diffeomorphic Anatomical Registration Through Exponentiated Lie Algebra (DARTEL) was used to create a custom study template by nonlinearly aligning gray matter segmentations [27]. Subsequently, native space gray and white matter images were spatially normalized to the template using the individual flow fields. The resulting gray matter images (isotropic 1.5 mm^3^ voxels) were modulated to preserve the total amount of gray matter from the native space image. Images were smoothed with an isotropic Gaussian filter of 6 mm full-width at half-maximum (FWHM). After processing, the quality of the segmentations was visually checked and none had to be excluded. Total intracranial volume (TIV) was calculated from segmented images in native space:

$$ \mathrm{TIV}=\mathrm{gray}\  \mathrm{matter}+\mathrm{white}\  \mathrm{matter}+\mathrm{cerebrospinal}\  \mathrm{fluid}. $$

To limit the analysis to gray matter voxels, a mask was created to include only voxels with a gray matter probability of 0.1, resulting in 311,613 voxels included in the analysis.

### Statistical procedures

Independent-sample *t* tests, Mann–Whitney *U* tests, or chi-square tests were used when appropriate to compare the groups on demographic and clinical variables using SPSS (version 22; IBM), with *p* < 0.05 considered statistically significant.

Prior to the imaging statistics, a linear regression was performed at each voxel to correct the gray matter volume for the effects of the nuisance variables age, gender, scanner, and TIV. Baseline cross-sectional gray matter differences between amyloid-positive and amyloid-negative subjects were examined using a general linear model. Proportional Cox hazards regressions to predict disease progression were performed at each gray matter voxel using the Coxphfit function implemented in MATLAB 2011a (MathWorks Inc.). The outcome measure was time to dementia onset. The independent variable was residual gray matter at each voxel. At each voxel, the residual gray matter volume was inverted and Z-transformed. The hazard ratio (HR) then represents the increased chance of progressing to dementia within the next time point per standard deviation decrease in gray matter volume. Proportional Cox hazards regression was also used to assess the hazard ratio associated with amyloid positivity in the whole sample and for continuous CSF Aβ_1–42_ values in the amyloid subgroups, while correcting for age and gender. Continuous amyloid measures were inverted so that HRs were directly comparable.

First, the voxel-wise Cox regression analysis was performed on the total sample. Next, analyses were repeated after stratification by amyloid status. Statistical significance was determined with nonparametric permutation tests [28]. The event or group label was reallocated randomly to each subject 10 times. For each of these permutations, the voxel-wise Cox regression was repeated. The results of all permutations at all voxels were pooled to sample the permutation distribution under the null hypothesis (=3,427,743 random tests). The 2.5th and 97.5th percentiles of this null distribution were used as the critical values for statistical significance representing a two-sided test with a probability of type I error of 0.05. Sampling of the null distribution by permutation testing was repeated for all subgroup analyses.

We tested the assumption of proportional hazards for each voxel-level test for the main analysis using Schoenfeld residuals [29] using the cox.zph function in R (R version 3.1.1; http://www.R-project.org) with survival package version 2.37-7. We found no more violations of the proportional hazards assumption than would be expected by chance (3.24% of tests were significant at *p* < 0.05).

## Results

Of the 276 subjects with MCI, 122 (44%) progressed to dementia. Among those who progressed to dementia, 104 (85%) subjects progressed to AD-type dementia and 18 (15%) subjects to another type of dementia (four fronto-temporal dementia, eight vascular dementia, one mixed vascular and AD, three Lewy body dementia, and two dementia unspecified). Clinical characteristics of subjects are summarized in Table 1. Subjects who progressed were on average older, had lower baseline scores on the Mini-Mental State Examination (MMSE), and had lower baseline CSF Aβ_1–42_ than subjects who remained stable. The groups had similar follow-up times. Subjects who were amyloid positive had a higher risk of progressing to dementia compared to amyloid-negative subjects (HR 2.4, *p* < 0.001). When stratifying for amyloid status, 160 subjects were amyloid positive and 99 (62%) of them showed clinical progression. Of those amyloid-positive subjects who progressed, most subjects progressed to AD-type dementia (n *=* 94, 95%). A total of 116 subjects were amyloid negative, of whom 23 (20%) subjects progressed. Amyloid-negative subjects more often progressed to non-AD dementias (57%) than AD-type dementia (43%). Within the amyloid-positive group, continuous CSF Aβ_1–42_ levels were unrelated to progression to dementia (HR 1.0, *p* = 0.8). Within the amyloid-negative group, continuous CSF Aβ_1–42_ levels were associated with an increased risk of progression to dementia (HR 2.2, *p* = 0.01).

**Table 1:** Subject characteristics according to progression and amyloid status

Characteristic	All subjects (*n* = 276)		Amyloid negative (*n* = 116)		Amyloid positive (*n* = 160)	
	Stable	Progression	Stable	Progression	Stable	Progression
Number of subjects	154 (56)	122 (44)	93 (80)	23 (20)	61 (38)	99 (62)
Age (years)	65.5 ± 7.7	68.3 ± 8.1*	63.8 ± 7.9	68.2 ± 8.7^†^	68.2 ± 6.5	68.3 ± 8.0
Male gender	97 (63)	67 (56)	64 (69)	16 (70)	33 (54)	51 (51)
Education	5.0 ± 1.5	4.9 ± 1.7	4.8 ± 1.7	5.0 ± 1.6	5.3 ± 1.2	4.9 ± 1.7
MMSE	27.0 ± 2.2	26.1 ± 2.6*	27.0 ± 2.2	26.6 ± 2.5	27.0 ± 2.2	26.0 ± 2.6^†^
CSF Aβ_1–42_	794 ± 307	534 ± 194*	999 ± 207	848 ± 172*	481 ± 104	462 ± 107
WMH (Fazekas)	0.99 ± 0.92	1.03 ± 0.80	0.88 ± 0.93	1.30 ± 1.02	1.15 ± 0.89	0.97 ± 0.73
NGMV	0.41 ± 0.04	0.39 ± 0.05*	0.42 ± 0.04	0.39 ± 0.06*	0.41 ± 0.04	0.39 ± 0.04^†^
Follow-up (years)	2.5 ± 1.5	2.6 ± 1.7	2.3 ± 1.4	2.8 ± 2.0	2.7 ± 1.7	2.6 ± 1.3
Follow-up diagnosis						
AD	–	104 (85)	–	10 (43)	–	94 (95)
DLB	–	3 (2)	–	2 (9)	–	1 (1)
FTD	–	4 (3)	–	3 (13)	–	1 (1)
VaD and mixed	–	9 (7)	–	6 (26)	–	3 (3)
Other	–	2 (2)	–	2 (9)	–	0 (0)

Data presented as mean ± SD or count (%)

*AD* Alzheimer’s disease, *CSF* cerebrospinal fluid, *DLB* Lewy body dementia, *FTD* fronto-temporal dementia, *MMSE* Mini-Mental state examination, *NGMV* normalized gray matter volume, *VaD* vascular dementia, *WMH* white matter hyperintensities measured with 4-point Fazekas scale

*p < 0.01 different from stable subjects

^†^ *p* < 0.05 different from stable subjects

### Brain regions predicting time to dementia

**Fig. 1:**
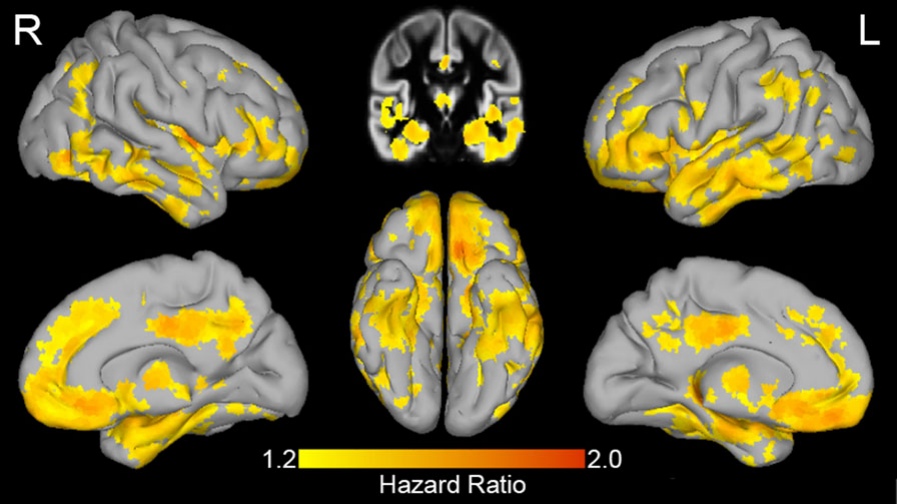
Brain regions predicting clinical progression in subjects with MCI. Hazard ratio for progression to dementia associated with lower residual gray matter volume depicted at each voxel. Residual gray matter volumes were standardized prior to analysis. R right, L left (Color figure online)

The voxel-wise proportional Cox hazards regressions showed that lower gray matter volumes in widespread cortical and subcortical areas were associated with time to progression to dementia (Fig. 1). In addition to decreased gray matter volume in well-known AD-related areas (i.e., hippocampal and temporal regions), low gray matter volume in the parietal and frontal regions was also associated with progression to dementia (Table 2). Repeating the analysis after excluding subjects progressing to non-AD dementia did not substantially change these results (data not shown).

**Table 2:** Regions in which decreased gray matter volume is associated with progression to dementia in subjects with MCI

Cluster size	MNI peak coordinates			Hazard ratio	Region
	*x*	*y*	*z*		
59,330	–9	30	–25.5	1.78	Left rectal gyrus
	43.5	1.5	6	1.78	Right insula
	–21	–34.5	–4.5	1.75	Left hippocampus
	27	–25.5	–15	1.70	Right parahippocampal gyrus
	57	–37.5	–7.5	1.69	Right middle temporal gyrus
	25.5	25.5	46.5	1.66	Right middle frontal gyrus
	–39	30	6	1.63	Left inferior frontal gyrus
	–54	0	–1.5	1.61	Left superior temporal gyrus
	1.5	42	–6	1.60	Right anterior cingulate
	16.5	42	34.5	1.59	Right medial frontal gyrus
2571	7.5	–31.5	40.5	1.67	Right cingulate gyrus
	10.5	–58.5	36	1.58	Right precuneus
797	39	–46.5	39	1.57	Right inferior parietal lobule
679	45	–70.5	–6	1.68	Right inferior temporal gyrus
540	–55.5	–54	36	1.42	Left supramarginal gyrus

Presented are the anatomical details of the five largest clusters. For the large clusters, several local maxima spread across the cluster are given

*MCI* mild cognitive impairment; *MNI*: montreal neurological institute space

### Influence of amyloid pathology

**Fig. 2:**
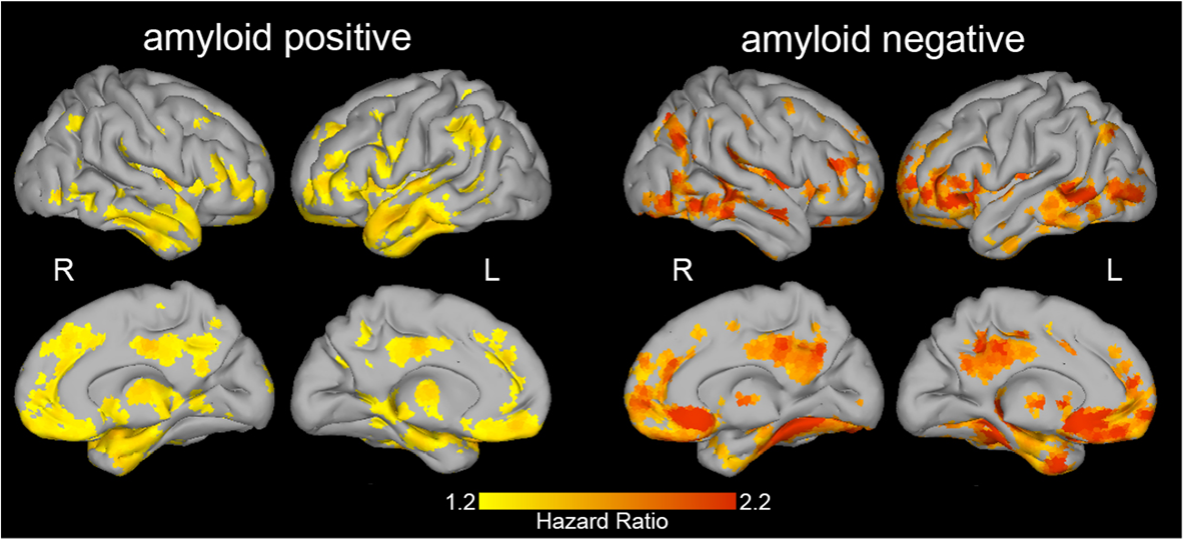
Brain regions predicting clinical progression in amyloid-positive and amyloid-negative subjects. Left panel: significant voxels predicting time to progression to dementia in amyloid-positive subjects. Right panel: significant voxels predicting time to progression to dementia in amyloid-negative subjects. Depicted are hazard ratios for progression to dementia, associated with lower residual gray matter volume. R right, L left (Color figure online)

First, we assessed the influence of amyloid status on gray matter volume with standard voxel-based morphometry. This revealed decreased gray matter volume in amyloid-positive subjects when compared to amyloid-negative subjects in the hippocampus, temporal, parietal, and frontal regions (Additional file 2: Figure S1). Amyloid-negative subjects had lower gray matter volume at baseline in the cerebellum than amyloid-positive subjects. Next, we repeated the survival analysis after stratifying the subjects according to amyloid status. Amyloid-positive subjects showed widespread decreases in gray matter volume that were predictive of time to progression to dementia, similar to the analysis in the whole sample (Fig. 2). In comparison, the anatomical patterns predicting progression in amyloid-negative subjects were qualitatively similar to those observed in amyloid-positive subjects, apart from bilateral anterior temporal regions (only significant in amyloid-positive subjects) and the right fusiform gyrus (only significant in amyloid-negative subjects). To formally assess the difference between amyloid-positive and amyloid-negative subjects, we added an interaction term between voxel gray matter volume and amyloid status to the voxel-level Cox regression on the whole sample. For the majority of voxels (97% of all voxels included in the analyses) this interaction was not significant, supporting that the predictive value of baseline gray matter volume is largely similar for amyloid-positive and amyloid-negative subjects (Additional file 2: Figure S2). Three somewhat larger clusters of voxels showed significant interaction effects of amyloid status: two ventromedial prefrontal regions, which showed a higher hazard ratio in amyloid-negative subjects compared to amyloid-positive subjects; and one cluster in the right temporal fusiform gyrus that was only significant in amyloid-negative subjects.

Because within the amyloid-negative group continuous CSF Aβ_1–42_ values were associated with progression to dementia, we performed a voxel-wise Cox regression after additionally correcting for continuous CSF Aβ_1–42_ values. This analysis resulted in HRs of similar size. When using a voxel-wise threshold of *p* < 0.005 a similar pattern of significant voxels emerged as in the analysis without correction for CSF Aβ_1–42_, but these did not survive correction for multiple testing (Additional file 2: Figure S3).

## Discussion

The main finding of this article is that a widespread pattern of decreased gray matter volume, beyond the hippocampal region, is predictive of time to progression to dementia in subjects with MCI. The presence of amyloid pathology was also a predictor of time to progression to dementia. The pattern of decreased gray matter volume that was predictive of progression was mostly similar in amyloid-positive and amyloid-negative subjects.

The cortical pattern of decreased gray matter volume predicting progression to dementia in subjects with MCI included the temporal, parietal, and frontal regions. Most of the voxels predictive of progression to dementia are located in brain regions that typically show atrophy and hypometabolism as a sign of neurodegeneration in subjects with AD [30, 31], and are known to be pathologically involved in AD [32]. Our results are largely in line with previous voxel-based morphometry studies comparing MCI subjects who progress to dementia with those who remain stable over time. These studies have identified the medial and lateral temporal lobe [13–15] and also the parietal and frontal areas [15] to be associated with progression. Only two studies have so far also applied voxel-level survival analysis to structural MRI data. Using this method, intersubject variability in rates of clinical progression and follow-up time can be taken into account. Those studies also reported that atrophy in (mesial) temporal and posterior cingulate structures is predictive of cognitive decline [16, 17]. The patterns of low gray matter volume predictive of decline observed in our study were more extensive than in these previous studies and additionally revealed the frontal and more widespread parietal regions to be involved. This could partially be explained by our higher power due to the larger sample size. Also, Zeifman et al. [17] examined clinical progression from a cognitively normal stage, whereas our sample comprised subjects with MCI at baseline, who have more atrophy and a higher probability to progress to dementia within a follow-up time of a few years.

The regional patterns of decreased gray matter volume predictive of progression to dementia in subjects with evidence of amyloid pathology were largely similar to those found in the whole sample, and were located in regions known to be associated with Alzheimer pathology. This is in line with the notion that subjects with MCI and evidence of amyloid plaques are at an early stage of AD [3]. In addition, we found that decreased gray matter volume in most of these regions was also associated with progression to dementia in MCI subjects with normal amyloid at baseline. This suggests that the regions of decreased gray matter volume we found to be predictive for time to dementia are not specific for amyloid pathology. These findings are in line with MRI studies using a priori defined areas, which have also found that brain regions typically atrophied in AD were predictive of cognitive decline in MCI subjects both with and without evidence of amyloid pathology [7, 9, 11]. We further extend those results by showing that a more widespread anatomical pattern of decreased gray matter volume is related to cognitive decline, independent of the presence of amyloid pathology.

A possible explanation for our results is that the subjects who progressed within the amyloid-negative group do have underlying AD pathology, but are above the cut-off point for amyloid positivity. CSF Aβ_1–42_ levels, although well above the threshold, were significantly lower in subjects who progressed than in those who remained stable (Table 1). Further characterizing the subjects who progressed within the amyloid-negative group revealed that those subjects who progressed to clinical AD type dementia also had lower baseline CSF Aβ_1–42_ than those who progressed to other dementias (740 ± 129 vs 930 ± 158, *p* < 0.01). Possibly, these subjects might have reached abnormal CSF Aβ_1–42_ levels at the time of dementia diagnosis (not tested), since several studies have shown that higher amyloid burden at baseline, even within the normal range, is associated with future amyloid depositions [10, 33, 34]. An alternative explanation is that subjects who progressed to clinical dementia in the absence of amyloid pathology are akin to the concept of suspected non-amyloid pathology (SNAP). This concept has been constructed for subjects who present with evidence of AD-like neurodegeneration in the absence of amyloid pathology [35]. We studied subjects with MCI, who suffer from cognitive impairment, suggesting that a neurodegenerative process has already caused neuronal damage, but the underlying cause might not be AD. There are several disorders that show a spatially overlapping atrophy pattern with regions that are typically affected in AD. For example, the medial temporal lobe is involved in hippocampal sclerosis and TDP43 pathology can be associated with widespread cortical atrophy [36–38]. The association between regional amyloid deposition in the brain and cortical atrophy is still unclear [32, 39]. Our results support the idea that some brain regions are selectively vulnerable to pathological factors in general, and that amyloid pathology is one possible initiator of a common neurodegenerative pathway [40].

Although the overall anatomical pattern of decreased gray matter volume associated with time to progression was largely similar in amyloid-positive and amyloid-negative subjects, some subtle differences can be appreciated in Fig. 2 and Additional file 2: Figure S2. In the ventromedial prefrontal cortex, HRs were higher in amyloid-negative subjects compared to amyloid-positive subjects. Furthermore, the right fusiform gyrus was only significantly associated with time to progression in amyloid-negative subjects. This region has previously been included in an AD signature cortical thickness measure [41]. Whether decreased gray matter volume in these regions could be an indication of non-AD pathology in subjects with MCI will need to be examined in larger studies.

A potential limitation of this study is that the data were acquired at a clinical center over a relatively long period of time, during which the dementia work-up protocols changed, diagnostic criteria evolved, and higher field-strength MRI scanners were implemented. Although we have corrected for the potential influences where possible, we cannot exclude the possibility that this variability might have led to an underestimation of our results. Still, this can also be considered a strong point of our study, as it supports the robustness of our results. Another limitation is that we defined the follow-up time when the clinical diagnosis changed as the outcome parameter for the survival analyses. Since follow-up times were not strictly standardized but rather based on clinical judgment and timing of a yearly appointment, this might have biased the results. However, we think this is unlikely since the time of follow-up, with an average of 2.5 ± 1.5 and ranging up to 11 years, was similar for subjects who progressed and those who remained stable.

## Conclusions

Widespread decreases in gray matter volume are useful for the prediction of clinical progression and time to dementia in subjects with MCI. Findings were largely similar in subjects with and without evidence of amyloid pathology. This leads us to consider that although brain atrophy does not seem specific for the underlying pathology, it is a useful marker that reflects incipient dementia and thereby is valuable for predicting clinical progression.

## Additional files

Additional file 1: Describes the MRI acquisition parameters for each of the scanners. (PDF 57 kb)

Additional file 2: Figures S1–S3. Examining the differences between amyloid-positive and amyloid-negative subjects. (PDF 2073 kb)

## Abbreviations

AD: Alzheimer’s disease
Aβ_1–42_: Beta-amyloid_1–42_
CDR: Clinical Dementia Rating
CSF: Cerebrospinal fluid
MCI: Mild cognitive impairment
MMSE: Mini-Mental State Examination
TIV: Total intracranial volume

## References

[1] Farias ST, Mungas D, Reed BR, Harvey D, DeCarli C (2009). Progression of mild cognitive impairment to dementia in clinic- vs community-based cohorts. Arch Neurol..

[2] Mitchell J, Arnold R, Dawson K, Nestor PJ, Hodges JR (2009). Outcome in subgroups of mild cognitive impairment (MCI) is highly predictable using a simple algorithm. J Neurol..

[3] Albert MS, DeKosky ST, Dickson D, Dubois B, Feldman HH, Fox NC (2011). The diagnosis of mild cognitive impairment due to Alzheimer’s disease: recommendations from the National Institute on Aging–Alzheimer’s Association workgroups on diagnostic guidelines for Alzheimer’s disease. Alzheimers Dement..

[4] Jack CR, Wiste HJ, Lesnick TG, Weigand SD, Knopman DS, Vemuri P (2013). Brain β-amyloid load approaches a plateau. Neurology..

[5] Villain N, Chételat G, Grassiot B, Bourgeat P, Jones G, Ellis KA (2012). Regional dynamics of amyloid-β deposition in healthy elderly, mild cognitive impairment and Alzheimer’s disease: a voxelwise PiB–PET longitudinal study. Brain..

[6] Villemagne VL, Burnham S, Bourgeat P, Brown B, Ellis KA, Salvado O (2013). Amyloid β deposition, neurodegeneration, and cognitive decline in sporadic Alzheimer’s disease: a prospective cohort study. Lancet Neurol..

[7] Da X, Toledo JB, Zee J, Wolk DA, Xie SX, Ou Y (2014). Integration and relative value of biomarkers for prediction of MCI to AD progression: spatial patterns of brain atrophy, cognitive scores, APOE genotype and CSF biomarkers. NeuroImage Clin..

[8] Vemuri P, Wiste HJ, Weigand SD, Shaw LM, Trojanowski JQ, Weiner MW (2009). MRI and CSF biomarkers in normal, MCI, and AD subjects: predicting future clinical change. Neurology..

[9] Dickerson BC, Wolk DA, Alzheimer’s Disease Neuroimaging Initiative (2013). Biomarker-based prediction of progression in MCI: comparison of AD signature and hippocampal volume with spinal fluid amyloid-β and tau. Front Aging Neurosci..

[10] Jack CR, Lowe VJ, Weigand SD, Wiste HJ, Senjem ML, Knopman DS (2009). Serial PIB and MRI in normal, mild cognitive impairment and Alzheimer’s disease: implications for sequence of pathological events in Alzheimer’s disease. Brain..

[11] Jack CR, Wiste HJ, Vemuri P, Weigand SD, Senjem ML, Zeng G (2010). Brain beta-amyloid measures and magnetic resonance imaging atrophy both predict time-to-progression from mild cognitive impairment to Alzheimer’s disease. Brain..

[12] Van Rossum IA, Vos SJB, Burns L, Knol DL, Scheltens P, Soininen H (2012). Injury markers predict time to dementia in subjects with MCI and amyloid pathology. Neurology..

[13] Karas G, Sluimer J, Goekoop R, van der Flier W, Rombouts SA, Vrenken H (2008). Amnestic mild cognitive impairment: structural MR imaging findings predictive of conversion to Alzheimer disease. Am J Neuroradiol..

[14] Risacher SL, Saykin AJ, Wes JD, Shen L, Firpi HA, McDonald BC (2009). Baseline MRI predictors of conversion from MCI to probable AD in the ADNI Cohort. Curr Alzheimer Res..

[15] Whitwell JL, Shiung MM, Przybelski SA, Weigand SD, Knopman DS, Boeve BF (2008). MRI patterns of atrophy associated with progression to AD in amnestic mild cognitive impairment. Neurology..

[16] Vemuri P, Weigand SD, Knopman DS, Kantarci K, Boeve BF, Petersen RC (2011). Time-to-event voxel-based techniques to assess regional atrophy associated with MCI risk of progression to AD. NeuroImage..

[17] Zeifman LE, Eddy WF, Lopez OL, Kuller LH, Raji C, Thompson PM (2015). Voxel level survival analysis of grey matter volume and incident mild cognitive impairment or Alzheimer’s disease. J Alzheimers Dis..

[18] Van der Flier WM, Pijnenburg YAL, Prins N, Lemstra AW, Bouwman FH, Teunissen CE (2014). Optimizing patient care and research: the Amsterdam Dementia Cohort. J Alzheimers Dis..

[19] Petersen R, Smith G, Waring S, Ivnik R, Tangalos E, Kokmen E (1999). Mild cognitive impairment: clinical characterization and outcome. Arch Neurol..

[20] McKeith IG, Dickson DW, Lowe J, Emre M, O’Brien JT, Feldman H (2005). Diagnosis and management of dementia with Lewy bodies: third report of the DLB Consortium. Neurology..

[21] McKhann G, Drachman D, Folstein M, Katzman R, Price D, Stadlan EM (1984). Clinical diagnosis of Alzheimer’s disease. Report of the NINCDS‐ADRDA Work Group under the auspices of Department of Health and Human Services Task Force on Alzheimer’s Disease. Neurology..

[22] Neary D, Snowden JS, Gustafson L, Passant U, Stuss D, Black S (1998). Frontotemporal lobar degeneration: a consensus on clinical diagnostic criteria. Neurology..

[23] Rascovsky K, Hodges JR, Knopman D, Mendez MF, Kramer JH, Neuhaus J (2011). Sensitivity of revised diagnostic criteria for the behavioural variant of frontotemporal dementia. Brain..

[24] Román GC, Tatemichi TK, Erkinjuntti T, Cummings JL, Masdeu JC, Garcia JH (1993). Vascular dementia Diagnostic criteria for research studies: report of the NINDS‐AIREN International Workshop. Neurology.

[25] Mulder C, Verwey NA, van der Flier WM, Bouwman FH, Kok A, van Elk EJ (2010). Amyloid-β(1–42), total tau, and phosphorylated tau as cerebrospinal fluid biomarkers for the diagnosis of Alzheimer disease. Clin Chem..

[26] Zwan M, van Harten A, Ossenkoppele R, Bouwman F, Teunissen C, Adriaanse S (2014). Concordance between cerebrospinal fluid biomarkers and [11C]PIB PET in a memory clinic cohort. J Alzheimers Dis..

[27] Ashburner J (2007). A fast diffeomorphic image registration algorithm. NeuroImage..

[28] Bullmore ET, Suckling J, Overmeyer S, Rabe-Hesketh S, Taylor E, Brammer MJ (1999). Global, voxel, and cluster tests, by theory and permutation, for a difference between two groups of structural MR images of the brain. IEEE Trans Med Imaging..

[29] Grambsch PM, Therneau TM (1994). Proportional hazards tests and diagnostics based on weighted residuals. Biometrika..

[30] Jack CR, Holtzman DM (2013). Biomarker modeling of Alzheimer’s disease. Neuron..

[31] Mosconi L, Tsui WH, Herholz K, Pupi A, Drzezga A, Lucignani G (2008). Multicenter standardized 18 F-FDG PET diagnosis of mild cognitive impairment, Alzheimer’s disease, and other dementias. J Nucl Med..

[32] Braak H, Braak E (1991). Neuropathological stageing of Alzheimer-related changes. Acta Neuropathol..

[33] Sojkova J, Zhou Y, An Y (2011). Longitudinal patterns of β-amyloid deposition in nondemented older adults. Arch Neurol..

[34] Villemagne VL, Pike KE, Chételat G, Ellis KA, Mulligan RS, Bourgeat P (2011). Longitudinal assessment of Aβ and cognition in aging and Alzheimer disease. Ann Neurol..

[35] Jack CR, Knopman DS, Chételat G, Dickson D, Fagan AM, Frisoni GB (2016). Suspected non-Alzheimer disease pathophysiology—concept and controversy. Nat Rev Neurol..

[36] Jack CR, Dickson DW, Parisi JE, Xu YC, Cha RH, O’Brien PC (2002). Antemortem MRI findings correlate with hippocampal neuropathology in typical aging and dementia. Neurology..

[37] Nelson PT, Smith CD, Abner EL, Wilfred BJ, Wang W-X, Neltner JH (2013). Hippocampal sclerosis of aging, a prevalent and high-morbidity brain disease. Acta Neuropathol..

[38] Whitwell JL, Jack CR, Parisi JE, Senjem ML, Knopman DS, Boeve BF (2010). Does TDP-43 type confer a distinct pattern of atrophy in frontotemporal lobar degeneration?. Neurology..

[39] Jack CR, Lowe VJ, Senjem ML, Weigand SD, Kemp BJ, Shiung MM (2008). 11C PiB and structural MRI provide complementary information in imaging of Alzheimer’s disease and amnestic mild cognitive impairment. Brain..

[40] Jagust W (2013). Vulnerable neural systems and the borderland of brain aging and neurodegeneration. Neuron..

[41] Jack CR, Wiste HJ, Weigand SD, Knopman DS, Mielke MM, Vemuri P (2015). Different definitions of neurodegeneration produce similar amyloid/neurodegeneration biomarker group findings. Brain..

