# Supplementary material for: Amyloid-independent atrophy patterns predict time to progression to dementia in mild cognitive impairment
Source: Alzheimers Res Ther. 2017 Sep 12;9:73. doi: 10.1186/s13195-017-0299-x (PMC5596472; doi:10.1186/s13195-017-0299-x)
Supplement: Supplementary file 1 — Describes the MRI acquisition parameters for each of the scanners. (PDF 57 kb) [file 13195_2017_299_MOESM1_ESM.pdf]

Amyloid-independent atrophy patterns predict time to progression to dementia in MCI

Mara ten Kate, MD; Frederik Barkhof, MD PhD; Pieter Jelle Visser, MD PhD; Charlotte E. Teunissen, PhD; Philip Scheltens, MD PhD; Wiesje M. van der Flier, PhD; Betty M. Tijms, PhD

Supplementary methods

MRI acquisition parameters

Subjects were scanned on 8 different scanners: 1T Siemens Magnetom Impact, 1.5T Siemens Vision, 1.5T Siemens Sonata, 1.5T Siemens Avanto, 1.5T GE SignaHDxt, 3T GE SignaHDxt, 3T Toshiba Titan and a 3T Philips Ingenuity PET/MR system.

The following acquisition parameters were used: Impact (MPRAGE): coronal plane, repetition time (TR) 15 ms, echo time (TE) 7 ms, inversion time (TI) 300 ms, flip angle (FA) 15°, voxel size 1×1×1.5 mm; Vision (MPRAGE): coronal plane, TR 15 ms, TE 7 ms, FA 8°, voxel size 0.98×0.98×1.5 mm; Sonata (MPRAGE): coronal plane, TR 2700 ms, TE 3.97 ms, TI 950 ms, FA 8°, voxel size 1×1×1.5 mm; Avanto (MPRAGE) coronal plane, TR 2700 ms, TE 5.2 ms, TI 950 ms, FA 8°, voxel size 1×1×1.5 mm; 1.5T Signa (FSPGR): sagittal plane, TR 12.4 ms, TE 5.17 ms, TI 450 ms, FA 12°, voxel size 0.98×0.98×1.5 mm; 3T Signa (FSPGR): sagittal plane, TR 8 ms, TE 3 ms, TI 450, FA 12°, voxel size 0.98×0.98×1 mm; Titan (FFE): sagittal plane, TR 9.5 ms, TE 3.2 ms, TI 800 ms, FA 7°, voxel size 1×1×1 mm; PET/MR (TFE): sagittal plane, TR 8 ms, TE 4 ms, FA 12°, voxel size 1×1×1 mm.
