# Supplementary material for: Amyloid-independent atrophy patterns predict time to progression to dementia in mild cognitive impairment
Source: Alzheimers Res Ther. 2017 Sep 12;9:73. doi: 10.1186/s13195-017-0299-x (PMC5596472; doi:10.1186/s13195-017-0299-x)

Amyloid-independent atrophy patterns predict time to progression to dementia in MCI

Mara ten Kate, MD; Frederik Barkhof, MD PhD; Pieter Jelle Visser, MD PhD; Charlotte E. Teunissen, PhD; Philip Scheltens, MD PhD; Wiesje M. van der Flier, PhD; Betty M. Tijms, PhD

Figure S1: Difference in brain atrophy between amyloid positive and negative subjects at baseline. In yellow-red are depicted voxels where amyloid positive subjects have decreased grey matter compared to amyloid negative subjects.

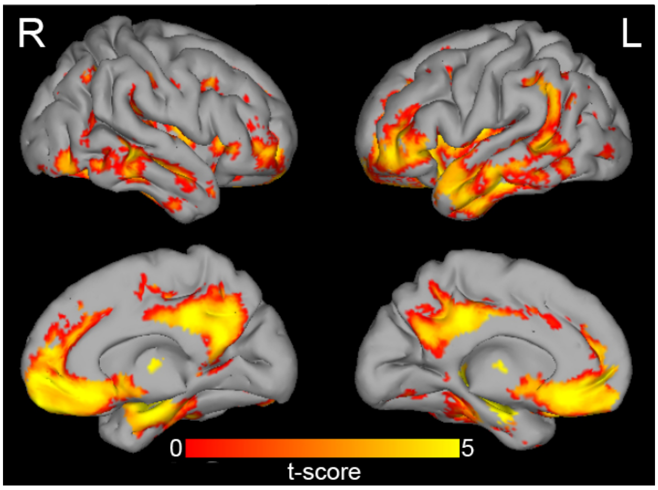

Figure S2: Difference in predictive value for clinical progression of grey matter volume between amyloid positive and amyloid negative subjects. Shown are voxels for which the interaction between grey matter volume and amyloid pathology for predicting clinical progression is significant.

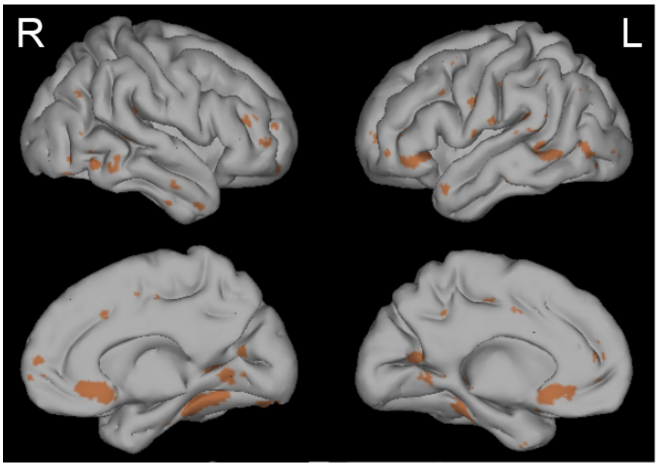

Figure S3: Hazard ratios for clinical progression after correcting for CSF A$\beta_{1–42}$ values in amyloid positive and negative subjects.

Hazard ratios for the voxel-wise Cox analysis with continuous CSF A$\beta_{1–42}$ as covariate. For both groups, the hazard ratios are comparable to the analysis without correction for CSF A$\beta_{1–42}$ (figure 2 main text). Displayed are voxels significant at a voxel-wise $p < 0.005$.

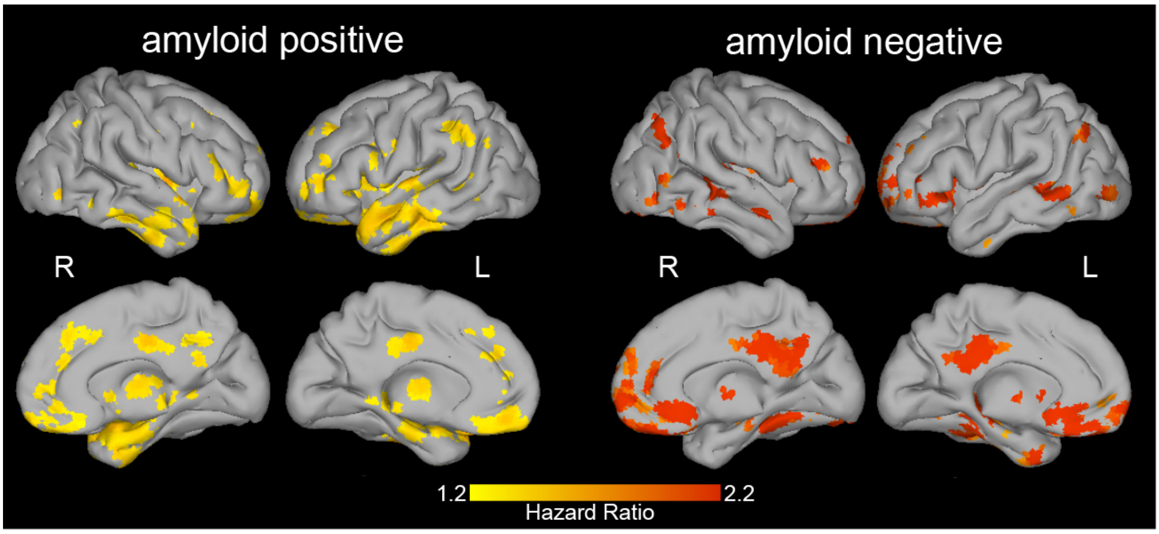

Supplement: Supplementary file 2 — Examining the differences between amyloid-positive and amyloid-negative subjects. (PDF 2073 kb) [file 13195_2017_299_MOESM2_ESM.pdf]
